# Young adults fall for non-democratic ideology regardless of their education and political leaning: a data report from a Czech physiological study

**DOI:** 10.3389/fpsyg.2023.1151226

**Published:** 2023-05-19

**Authors:** Martin Petlach, Michal Ondruška

**Affiliations:** ^1^Department of Territorial Studies, Faculty of Regional Development and Territorial Studies, Mendel University in Brno, Brno, Czechia; ^2^Institute of Political Sciences, Faculty of Social Sciences, Charles University, Prague, Czechia

**Keywords:** ideology, political leaning, electrodiagnosis (EDX), fEMG, EDA, education

## 1. Introduction

The number of countries, identifying themselves as liberal democracies, have decreased recently after yielding to various forms of electoral authoritarianism due to the citizens' characteristics and political attitudes (Morse, 2012; Schedler, 2015; Freedom House, 2019). At the same time, the outbreak of the COVID-19 pandemic also exacerbated the living conditions for democracy (Moscatelli et al., 2023). Therefore, natural sciences and psychology have gradually altered how political behavior is approached (Jost et al., 2014a). Consequently, biological science has become an indispensable fixture of political science (Smith et al., 2011; Hatemi and McDermott, 2012; Schreiber, 2017). New studies, originally arising from political science, have been expanded on by psychologists in the context of Central and Eastern Europe (CEE). The local studies then conclude that the democratic nature of given societies keeps dropping (Klicperová-Baker, 2021). Especially in the case of CEE, democratic backsliding has been recognized as the process of “de-consolidation” (Bochsler and Juon, 2020, pg. 167).

Consequently, the authors address the importance of physiology and physiological reactions within political psychology in two types of electrodiagnosis (EDX) experiments while the authors simultaneously recorded electrodermal activity (EDA), measuring the skin conductance responses (SCR), and the facial muscle activity via facial electromyography (fEMG). The authors attempt to (1) partially elaborate on the discrepancy between Amodio et al. (2007) and Kremláček et al. (2019) whose teams analyzed the role of political leanings with different conclusions, and (2) study non-democratic ideology and its potential devotees as their numbers snowball at an alarming rate. Whereas Amodio's study, based on event-related potentials (ERPs), gave evidence of political leaning (conservative or liberal) as a key variable linked to one's brain activity, Kremláček's EEG experiment suggested the opposite thereby identifying no relation between political leaning and the brain activity of their non-Western research participants. Another objective of this study is to call attention to EDX as an approach for studying long-term aspects of political behavior (Oxley et al., 2008), rather than only short-term ones (Klofstad, 2017). However, the tools and methods of EDX and the level they attest to the overall relevance to social science have neither been sufficiently analyzed nor confirmed, for instance, in the case of EDA (cf. Ravaja, 2009; Leiner et al., 2012; Horesh et al., 2021) or fEMG (cf. Jerritta et al., 2014; Isabella et al., 2015; Drimalla et al., 2019). The authors outline feasible trajectories of research in which self-identification and questionnaires would not be the only indicator (cf. Innes and Ahrens, 1994; Erisen et al., 2013).

Nevertheless, the current number of studies employing electrodermal activity (EDA) in connection to politics is more than restrained. Notwithstanding its potential (see Johnson et al., 2010), fEMG has been mostly omitted (Hibbing et al., 2014a). In the few studies examining EDA, researchers usually use aversive and appealing photographs to differentiate political leanings among examined participants. Their conclusions (Oxley et al., 2008; Dodd et al., 2012) put the accent on the fact that conservatives pay significantly more attention to aversive photographs which elicits [their] greater physiological reactions. Conversely, liberals instead concentrate on agreeable photographs. Oxley et al. (2008) put this aspect into the context of brain neuroanatomy when stressing the role of the amygdala. Accordingly, they summarize that political orientation is conditioned by physiological predispositions.

## 2. Expectations

The authors focus on political leaning and the type of education as they compare young adults, specifically the pupils with the prime quality education of high schools, and their counterparts attending a secondary vocational school which puts less emphasis, in general, on general knowledge in the Czech Republic. Education has already been confirmed as an influential variable affecting political behavior and attitudes (Gallego, 2010; Ansell and Lindvall, 2013; Baker and Whitehead, 2015), and similarly ideology in the form of political leaning runs through the papers as a key indicator (Jost et al., 2008; Dodd et al., 2012; Weissflog et al., 2013; Mills et al., 2016; Kalmoe and Johnson, 2022).

In practice, the authors investigate whether the groups of young adults reacted differently to non-democratic ideology and whether their political leaning and education could have been linked to those physiological reactions. The authors investigate into significantly positive physiological reactions symbolizing non-democratic leaning. Even though the collocation itself, as well as non-democratic tendencies, is usually attributed to the theories of international relations (Kästner, 2010; Bieling, 2012). The authors propose the following hypotheses:

H1: The participants representing two different types of education will exhibit divergent physiological traits in response to non-democratic ideology.

H2: The participants representing two different types of education will exhibit divergent physiological traits depending on their political leaning.

## 3. Methods

### 3.1. Participants and procedure

The experiment started between 9 March 2020 and 10 March 2020 with the first set of participants, and another set of participants took part in the experiment on 21 April 2020. In total, there were 10 participants in this pilot experiment.^1^ While five participants were pupils of two prestigious high schools (G) from the Czech Republic, the other five participants were pupils at a secondary vocational school (S). The participants were randomly selected, only if they just turned 18 years of age, and declared no history of neurological or psychiatric diseases. Due to their recent coming of age, all the participants were entitled to sign the informed consent form.

To assess the participants' reactions to ideologies, it was imperative that they would have reacted not only to the appearance of selected leaders but also to the ideology. For this reason, the participants had been emailed with a pdf file containing all the photographs with legends, which consisted of positive and negative types of information too, a week beforehand, so that they were given enough time to go through them. While dealing with the technical and introductory aspects of the experiment (e.g., the informed consent), the authors asked about the content of the pdf in passing before the actual and formal double-checking as described in the following. In this matter, it is necessary to point out that especially non-democratic ideologies are being personalized through their leaders as they signify the core of ideological doctrines (Sartori, 1995).

In the laboratory, the authors asked the participants to watch 30 photographs.^2^ While 10 of the photographs portrayed democratic politicians, the other 10 embraced non-democratic politicians, and 10 photographs were neutral. Every photograph was displayed one by one for 8 s during when the participants were asked to recall and visualize all the information. After answering a simple question (What can you tell us about this person?), a scale ranging between 0 and 2 could be employed in which 0 represented no association and knowledge of the person, 1 represented basic knowledge, 2 represented a complete awareness that included the person's opinion and ideological background. This scale was then used to monitor and double-check the participants' knowledge and their overall acceptability for the analysis. Afterward, an additional scale ranging from 1 to 7 appeared while asking the participants (How much would you agree with the attitudes of this person?) about their points of view. On this scale, 1 represented “Strongly disagree,” 4 represented “Non-applicable (N/A)” as the participant was not able to identify the person, and 7 represented “Strongly agree.”

During the experiment, electrodermal activity together with positive and negative responses were recorded, namely, through the zygomaticus major and *corrugator supercilii*. The authors then followed the standardized approach of Hibbing et al. (2014b) when surveying the participants' political leaning. With respect to programing and the experimental procedure, the authors used the AcqKnowledge software followed by two amplifiers—EMG 100C and GSR 100C—for recording the muscle to acquire all the raw data. it was the electrical activity and electrodermal activity and skin conductance, respectively, operating with the constant voltage of 0.5 V, and which had formerly been termed galvanic skin response (GSR).

### 3.2. Data procedure

Recordings of facial electromyography (fEMG) are mostly disrupted by eye movements, such as blinking, which may entail spurious deflections. This frequency noise can be avoided if the electromyogram is filtered. There are two types of frequency noise, high or low. And the latter appeared in the recordings of this experiment. While the high-frequency noise is addressed by the low-pass filter, the low-frequency noise, conversely, is fixed by a high-pass filtering that allows removing of the lower frequencies, and therefore, the overall recording is smoothened. However, according to van Boxtel (2010), it is essential to employ both filters so that the frequency ranges between 20 and 500 Hz, which means that the frequency is well within the “bandpass.” This double-ended filter was the first step out of the three filters in the data adjustment. Second, the data had to be transferred into absolute values. Every average rectified value (ARV) was achieved after overturning the values from the bottom up. Third, integrals had to be assigned for each photograph per participant to detach the knoll of activity.

Electrodermal activity (EDA) and its recordings are largely affected by the heartbeats. First, the low-pass filter at 0.05–1 Hz had to be used so that the repetitious waves, in the frequency of one time per 20s, could be suppressed. Second, it was key to adjust the time slot in the recorded data because the electrodermal activity is invariably 1 s delayed. Hence, out of 8 s when the photographs had been displayed, the first second had to be excluded. Third, the regular SCR range within the scope of 0.1–1 s. Reactions of the values under 0.1 s had to be restrained since there had been no reflection of the photograph.^3^ Finally, there were participants whose reactions were vitiated, and thus could not be used for the analysis.^4^ It implies that those participants who had been pondering upon something different, for example, moved their legs significantly or scratched their faces. Afterward, the logarithms for all the obtained figures had to be calculated for the purpose of normalization. For the statistical processing, the participants were treated as additional factors, meaning that everyone was assorted to 30 rows, thereby serving as fixed variables. For this reason, linear regression could not be used and it had to be substituted by the mixed-effect model, calculated in R, and visualized via coefplot. Boxplots with jitter pictorialize separate observations.

### 3.3. Data characterization

**Figure 1:**
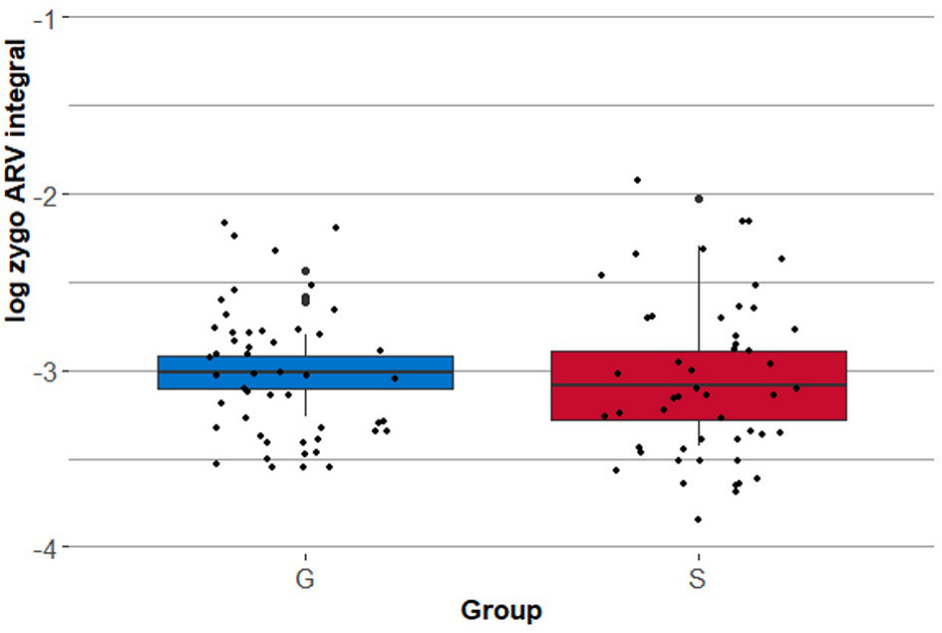
Positive physiological reactions to non-democratic ideology.

The authors detected a significant similarity in positive reactions via the zygomaticus major to non-democratic ideology from both examined groups. Figure 1 shows that the groups of pupils that differentiate neither in the distribution nor in the allocation of outliers. After running the independent *t*-test for equality of means (scoring 0.587), the authors conclude that there is no significant difference between the two groups.^5^

**Figure 2:**
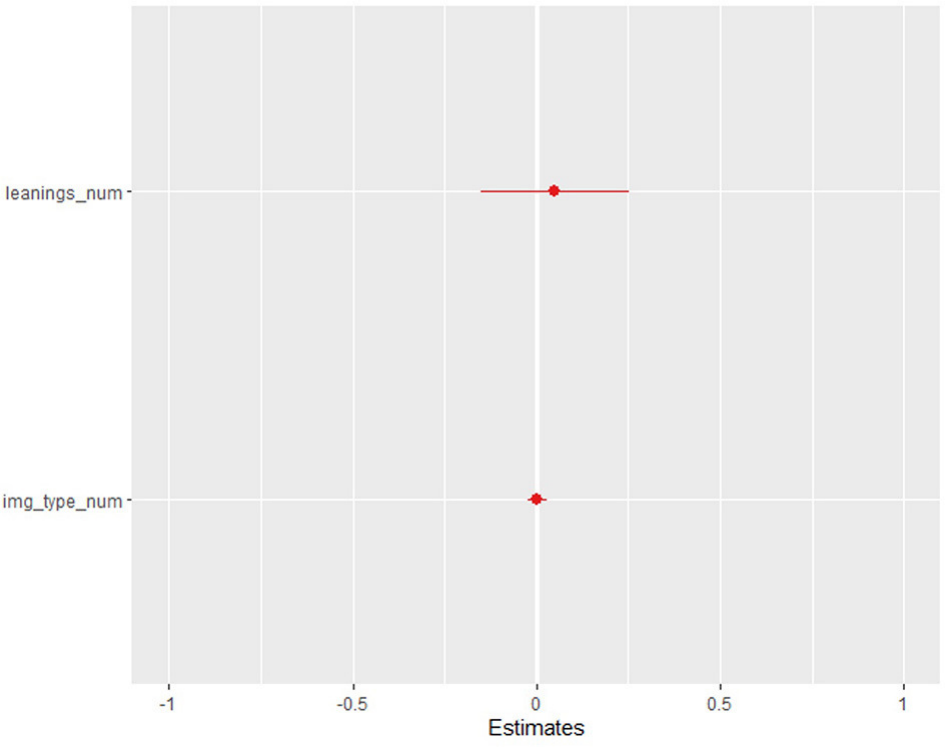
Positive (zygomaticus major) reactions and political leaning.

In the fixed-effect model and *t* > 0.05, the authors verified no effect of ideological leaning as a variable. In the random-effect modeling, the authors testified to the absence of significance regarding any liaison between the positive and/or negative responses and political leaning as depicted in Figure 2. Similarly, the figures of the fixed-effect model scored *t* > 0.05, thus showing no effect. When considering the data from the fixed-effect models in the case of political leanings, no significance has been verified and therefore, the variance in the models could not be elucidated by this variable. Overall, it implies that, on average, there is a difference between these two examined groups of pupils in their physiological reactions to different ideological poles, but the variable of political leaning has elucidated neither the divergence in physiological responses from the zygomaticus major, *corrugator supercilii*, nor the skin conductance response (SCR). Needless to emphasize that due to the indicative character of results caused by the number of participants, other variables, such as party preference and participants' gender, have not testified to be germane indicators.

At the same time, there was no salient divergence between the two groups in their SCR neither in dispersion nor in average. The authors also conclude that the SCR cannot be verified as a sufficient factor pertaining to political leaning. The same situation has been exemplified by the presence of no effect in the random- and fixed-effect models. The above-mentioned physiological responses were contrasted with the variable of one's approval of non-democratic ideology within the 1–7 scale. Non-democratic ideologies, nonetheless, had acquired a conspicuously higher approval from the examined group of secondary vocational school pupils (s) when scoring with a substantial significance.

## 4. Discussion

The authors endeavored to examine whether the pupils with their prime education fall for non-democratic ideology as easily as their counterparts with low-quality education, and whether political leaning may play any role in this situation. For this pilot study, physiological reactions were recorded through facial electromyography (fEMG) and electrodermal activity (EDA) to address the hypotheses. Although the examinations resulted in a restrained manner of generalizability, which is in line with the EEG study of Kremláček et al. (2019), the authors did not verify any liaison between political leaning and the physiological data recorded from the Czech participants. Therefore, political leaning may not necessarily represent such a strong variable, as in the West where Amodio et al. (2007) had initially conducted their pioneering experiment. Not only do the results from this physiological experiment confirm the problematic character of political leaning as a variable outside the Western countries, as propounded by Kremláček et al. (2019), but they also show that education may be less explanatory as a variable in terms of getting enthusiastic about non-democratic ideologies, notwithstanding the level of pupils' self-declared approval of non-democratic ideology. This brings back Kerlinge (1984) contention when he stated that ideology per se was solely “important in the Western world.” Given the era of that assumption, the question arises is “how relevant the variable of ideology could ever be in CEE?”

When analyzing respective ideologies and doctrines, it is traditionally just conservatism being perceived as the aberrant one (cf. Wilson, 1973; Bhattacharya, 2007; Etchezahar and Brussino, 2013). Since the 1950s and “The Authoritarian Personality,” conservatism has been associated with authoritarianism, even though this hypothesis has already been falsified (Roets and van Hiel, 2006; Stenner, 2009). The general belief is that representatives of conservatism and liberalism as two key positions differ in many aspects, such as cognitive skills and information processing (see Amodio et al., 2007). However, another feature peculiar to the area of CEE is an extensive inclination to and importance of political centrism (Petrović et al., 2022). The question is whether non-democratic or authoritarian tendencies may also be associated with centrist attitudes as occurring in CEE. Lindgren (2012), for example, pondered upon the existence of “centrist authoritarianism.” His extensive study discovered that, in American politics, those who had identified themselves as moderate or centrist tended to score significantly higher in respective psychological tests, thereby implying a greater scale of authoritarian tendencies. Conversely, “regular” conservatives and liberals exhibited saliently lower figures. It is necessary to emphasize that in CEE, authoritarianism has also been detected within the left-leaning spectrum or on both poles of the left–right scale (De Regt et al., 2011; Aspelund et al., 2013; Conway et al., 2022).

Hatemi and McDermott (2012) felicitously foreground that the overall complexity of this type of research is being amplified by the “chicken-and-egg problem.” This issue questions where the original and primary incentives as triggers really reside—whether in biological traits, traditionally in the human brain, or ideology or elsewhere (cf. Jost et al., 2003, 2014b; Feldman and Huddy, 2014; Hibbing et al., 2014b). Another interconnected aspect exacerbating the situation lies in political cognition, a subcategory that was identified within social cognition. Owing to the complex character of this variable, political cognition may then resemble “riding a bicycle.” Because of that, it is not easy to determine the factors affecting political thinking and behavior since “[people] don't know what they don't know” (Lieberman et al., 2003, p. 682). Due to this reason, scholars have not adequately recognized the roots and progress of political cognition (Edwards, 2003).

One of the limitations of the study to consider may be the number of participants in this pilot study in comparison to traditional studies which usually examined political cognition and were based on questionnaires (e.g., Richardson, 1998). Regardless of the use of physiological data, the overall generalizability may then be further enhanced by either recurring measurements in a diachronic perspective, increasing the number of participants, or expanding on and particularizing the socioeconomic status of those taking part in the experiment. All of these may come more costly, though especially in comparison to questionnaire surveys. Importantly, this study attempted to highlight the use and potential of EDX for political psychology. It is necessary to differentiate between two types of investigated phenomena: long-term patterns of political behavior (e.g., conservatism and its links to authoritarianism) vs. short-term patterns of political behavior (e.g., political campaigning and its effect or voting behavior), which usually experience swift changes and yield to swing voters. The authors anticipate that the use or amalgamation of EDX and additional political analyses may gradually become all the rage.

Although the era of ideologies has not ceased to exist (Jost, 2006), it emerged that its understandings vary distinctively in the post-Soviet countries (Thorisdottir et al., 2007). Additionally, this study stressed the far-reaching influence of non-democratic ideology, regardless of the participants' education and political leanings. However, it has left enough room for further research in, for example, emotions in political processes as a less traditional research topic in Europe (see Schreiber, 2017) as well as voting patterns.

## Data availability statement

The datasets presented in this study can be found in online repositories. The names of the repository/repositories and accession number(s) can be found in the article/supplementary material.

## Ethics statement

This study involving human participants was reviewed and approved by the Ethics Commission of Department of Psychology, Palacky University, Czech Republic. Written informed consent to participate in this study was provided by the participants.

## Author contributions

MP was a principal investigator (research design, data collection, supervision, and review). MO provided formal data analyses and feedback on the data. All authors contributed to the article and approved the submitted version.

## Footnotes

^1^Three extra participants were used for the preliminary testing. Although the findings and raw data from those participants were not part of the analysis, their role had been crucial for finalizing the research methods and programming the software.

^2^The original idea was to use 60 photographs, but this number had to be decreased to avoid habituation.

^3^Instead, it was a non-specified SCR (NS-SCR).

^4^Out of 300 examined cases, only one participant's set of SCR reactions (30) was found inconclusive as were few other individual reactions (32) from other participants.

^5^Additional data and graphs can be found in an online repository at the following link: http://doi.org/10.17605/OSF.IO/6ZYKS.
